# Integrated Community Care Delivered by Public Health Care and Social Care Systems: Protocol for a Realist Synthesis

**DOI:** 10.5334/ijic.5629

**Published:** 2021-10-26

**Authors:** Jean-François Allaire, Yacine Thiam, Paul Morin, Hervé Tchala Vignon Zomahoun, Nathalie Rheault, Francis Lacasse, Chantal Doré, Shelley-Rose Hyppolite, Suzanne Garon

**Affiliations:** 1 Institut universitaire de première ligne en santé et services sociaux (IUPLSSS) du Centre intégré universitaire de santé et de services sociaux de l’Estrie – Centre hospitalier universitaire de Sherbrooke (CIUSSSE-CHUS). Hôpital et centre d’hébergement D’Youville. 1036 Belvédère Street S. Sherbrooke, Qc, Canada. J1H 4C4, CA; 2 Institut universitaire de première ligne en santé et services sociaux (IUPLSSS) du Centre intégré universitaire de santé et de services sociaux de l’Estrie – Centre hospitalier universitaire de Sherbrooke (CIUSSSE-CHUS). Professor, School of Social Work. Université de Sherbrooke, CA; 3 Knowledge Translation Component of the Quebec SPOR-SUPPORT Unit, CA; 4 Centre intégré universitaire de santé et de services sociaux de l’Estrie – Centre hospitalier universitaire de Sherbrooke (CIUSSSE-CHUS), CA; 5 Université de Sherbrooke. Researcher, Institut universitaire de première ligne en santé et services sociaux (IUPLSSS) du Centre intégré universitaire de santé et de services sociaux de l’Estrie – Centre hospitalier universitaire de Sherbrooke (CIUSSSE-CHUS), CA; 6 Centre intégré universitaire de santé et de services sociaux de la Capitale nationale. Associate Professor, Faculty of Medicine, Université Laval, CA; 7 Université de Sherbrooke. Researcher, Institut universitaire de première ligne en santé et services sociaux (IUPLSSS) and Centre de recherche sur le vieillissement du Centre intégré universitaire de santé et de services sociaux de l’Estrie – Centre hospitalier universitaire de Sherbrooke (CIUSSSE-CHUS), CA

**Keywords:** Integrated community care, health care, social care, public services, proximity, local area

## Abstract

**Introduction:**

Integrated Community Care (ICC) is defined as an interweaving of territory scale and time scale health care and social care interventions implemented in proximity (spatial and relational) in an interdisciplinary and cross-sectoral manner. However, the deployment of in public health and social care networks can be complex owing to their broad mandate and the complexity of their management and accountability. Therefore, we aim to describe integrated community care in order to shed light on how they work, for whom and in what circumstances.

**Theory and methods:**

We will conduct a realist synthesis to design a flexible and scalable theory of the functioning of ICC deployed by public health and social care networks. To do so, a two-phase approach will be used: a systematic review on the topic of interest; and co-development and refinement of theory with local and international stakeholders. This data will be analyzed using both qualitative and quantitative methods.

**Dissemination of results:**

The results will be disseminated through peer-reviewed publications, academic presentations and a policy brief. This last document will include evidence on how ICC can be deployed by public health and social care networks to produce the impacts targeted.

## Introduction

Integrated Community Care (ICC) is “an interweaving of localized and temporalized health care and social care interventions provided in proximity (spatial and relational) in an interdisciplinary and cross-sectoral manner. ICC aims to improve physical and mental health, well-being and empowerment, as well as to facilitate access to and use of care, particularly among disadvantaged populations or those not served by the health and social care system.” [1].

ICC can be distinguished from conventional care by the four distinctive features of its delivery:

1. The approach used: health care, social care or integrating health and social care;
2. The environments that deploy them: the public health and social care network, the non-profit and community sector, the municipal sector, the private sector, or through the collaboration of these sectors;
3. The targeted population: disadvantaged communities, addressing all or major groups of the population (e.g., seniors, families, residents of social housing complexes) of a local area that is home to vulnerable or marginalized populations (material or social disadvantages), while specifically taking into account populations at the margins of the health and social care system;
4. The outcomes pursued: improving the health and well-being of individuals and communities, health equity, social capital within the community, social networks, social cohesion, and participation in co-production; improving accessibility, availability and continuity of health and social care; and taking action on social determinants of health.

In public health and social care networks, the deployment of ICC can be a complex task, owing to the types of outcomes being pursued but also the type of management and accountability that ICC require [2,3]. Indeed, in terms of management, the public network tends to be centralized [4] while ICC favours what Levesque, Harris, and Russell [5] call decentralized, collaborative, citizen governance. In terms of the type of accountability, the objectives pursued by ICC tend to require qualitative indicators (improvement of well-being, social capital, etc.) focused on “meaning,” whereas public networks that conduct government action generally focus on “measurement” [5], requiring quantitative indicators (number of interventions, number of users, duration and frequency of intervention, etc.). Beyond the complexity of its deployment, ICC is considered complex because the outcomes anticipated following deployment are neither constant nor systematically observable [1]. Indeed, ICC outcomes can vary according to the context of deployment (types of environments, types of stakeholders, and types of approaches), the characteristics of individuals, groups and populations, but also the characteristics of the territory and the temporality.

Thus, in order to better understand ICC and to encourage ICC practice in public health and social care networks, an international realist synthesis is being conducted to document their functioning and analyze the processes that produce the outcomes they target. With this in mind, this paper describes the protocol of this research conducted by a research team and an advisory committee made up of managers, practitioners, users and researchers affiliated with several institutions and programs.

## Choice and justification of the type of review carried out

Realist synthesis was chosen because it offers an approach adapted to the analysis of complex fields [6]. Inspired by critical realism, the realist approach [7] proposes a way of understanding the complexity of ICC-type interventions by seeking to understand how they work, for whom and in what contexts. Considering social interventions as theories [8], the realist approach does not aim to produce general theories in order to identify the most effective type of intervention in any given context [9]. Rather, it considers that the intervention is not the device that produces the outcomes but rather the mechanism or the way in which the actors interpret the logic of the intervention that produces them. This use of the term “mechanism” should not be confused with the general use of the term in program evaluation, which refers to the activity or mode of operation of the program being evaluated [10]. In a realist approach, mechanisms are the way in which the actors (stakeholders as well as beneficiaries) use the resources made available by the intervention to achieve the change targeted by the intervention [11]. These mechanisms are sensitive to variations in context and are generally not directly observable [12]. Given that the outcomes expected following the deployment of ICC are neither constant nor systematically observable, the realist approach aims to identify demi-regularities, namely the…

“regular, but not necessarily permanent, occurrence of an outcome following an intervention that triggers one or more mechanisms in a particular context […], thereby expressed by Context-Mechanism-Outcome configurations” (free translation from French). [6, p. 88].

Thus, the realist approach allows a theorization of Context-Mechanism-Outcomes configurations (CMOc) and the production of a middle-range theory (Middle-Range Theory) [13]. A middle-range theory is a level of theoretical abstraction that aims to establish a theory that is contextual and sufficiently explanatory to understand the functioning of the observed intervention with respect to targeted individuals or populations [6,7,14]. Conversely, a general theory makes it possible to predict the outcomes of a social intervention on individuals or targeted population regardless of the context. The realist synthesis presented in this paper therefore aims to design a flexible and evolving model of the operations of ICC deployed by public health and social care networks.

## Research question

Based on the PICOSS (Population/Intervention/Comparison/Outcome/Study design/Setting) approach of Cochrane systematic reviews [15], the research question for this realist synthesis is as follows: *How, why, for whom, and in what contexts does ICC deployed by the public network operate and produce outcomes?*

## Methods

We registered the present protocol for a realist synthesis on Open Science Framework under the hyperlink *osf.io/5wy2e*. We adapted the Preferred reporting items for systematic review and meta-analysis protocols (PRISMA-P) 2 [16] to draft our protocol.

**Figure 1:**
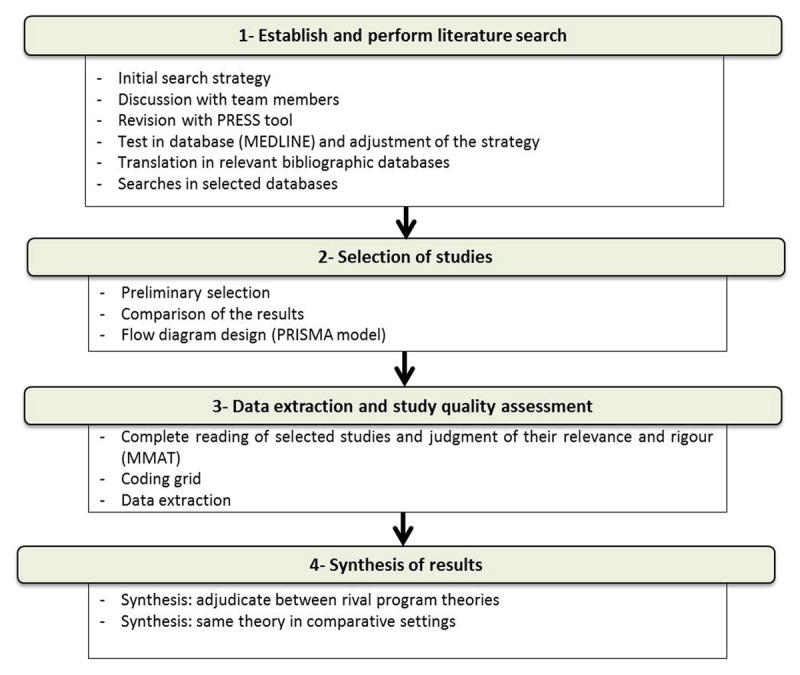
Systematic review operations.

The present protocol for a realist synthesis is divided into two steps: systematic review followed by the development of middle-range theories. The systematic review will be carried out in four stages. ***Figure 1*** presents an overview of the operations to be put in place to carry it out. The steps are described in greater detail below.

### Establish and perform literature search strategy

The literature search strategy involved two information specialists as well as the research team.

#### Criteria for inclusion and exclusion of studies

To be included in the review, a publication must be an empirical study of an ICC. The main criteria for inclusion of studies are (***Table 1***):

- addressing localized community-based health care, localized community-based social care or both integrated at the local area level, local area defined as “a socio-spatial entity, a living environment strongly shaped by its inhabitants, their interpersonal and social dynamics, their demographic characteristics, their history and their culture.” [1]. It is neighbourhoods and villages that refers to geographic, lived, perceived and designed local area.
- addressing all or major groups of the population (e.g., seniors, families, residents of social housing complexes) of a local area that is home to vulnerable or marginalized populations (material or social disadvantages), while specifically taking into account populations at the margins of the health and social care system. Specific subgroups of the population such as people with HIV or veterans restrict the target of the intervention and do not allow to act on targeted outcomes such as social capital increase. These papers will therefore be excluded.
- being deployed by public health or social care networks, with or without the collaboration of community or private partners.

**Table 1:** Definition of inclusion criteria following the PICOSS approach.


RESEARCH QUESTION COMPONENT	CHARACTERISTICS	DEFINITIONS

**Population**	Description, characteristics	– all or major groups of the population (e.g., seniors, families, residents of social housing complexes) – that is home to vulnerable or marginalized populations (material or social disadvantages) within specific local areas

**Interventions**	Content and terms of delivery	– Localized community-based health care – Localized community-based social care – or both integrated at the local area level – deployed by public health or social care networks, with or without the collaboration of community or private partners

**Comparator**	Common alternatives	– Regular health care and social services

**Outcome(s)**	Types of results oroutcomesproduced	– Improved health and well-being of individuals and communities – Improved health equity – Improved community social capital, social networks, social cohesion – Participation in co-production – Improved accessibility, availability and continuity of health care and social care – Action on social determinants of health

**Setting**		– Public providers – Local area


Excluded from the review are any studies or publications that present ICC deployed solely by non-profit or private sectors.

#### Data sources

The following bibliographic databases, enabling literature indexing and providing access to abstracts will be consulted: Medline (Ovid), Embase (*embase.com*), CINAHL (EBSCO), ÉRUDIT, CAIRN, PsycINFO (Ovid), Sociological Abstracts (Proquest), Web of Science.

#### Search strategy

The search strategy will be designed using controlled or free vocabularies derived from some elements of PICOSS (see Appendix 1). The preliminary version of the search strategy will be produced in Medline by an information specialist. Afterwards, in an iterative manner and with the collaboration of another information specialist, the search strategy will be refined, tested and discussed with the members of the research team and the advisory committee. The second information specialist will revise the preliminary search strategy using the Peer Review of Electronic Search Strategies (PRESS) tool [17].

The search strategy targets academic papers, books, and other publications related to the ICC theme and published in the period 2003–2019. The decision to begin collecting data in 2003 was justified by the publication of the second edition of Social Determinants of Health: The Solid Facts [18], which represented a major turning point in thinking about decision-making issues in the development of public action on the social determinants of health. In addition, since then, the World Health Organization (WHO) has undertaken several actions on these issues (Commission on Social Determinants of Health [19], Resolution WHA62/R14 [Sixty-second World Health Assembly, 2009], Adelaide Statement on Health in All Policies – [20], etc.).

#### Perform literature search

Two categories of publications will be considered: academic publications and publications from the grey literature.

1. Academic papers will be mainly searched by free and controlled vocabularies in the bibliographic databases mentioned above. Any relevant publication on the subject will be retained regardless of the language of publication.
2. Grey literature includes print or electronic publications on the subject of ICC but not published by academic journals publishers. This type of literature generally comes from government, academic, community, or private publications. In addition, it should be noted that the research team will be able to call upon its international network of contacts (mainly based in Canada, Italy, England and Scotland) to supplement information in order to identify other documents relevant to the review, should the need arise. Five researchers who have had past collaborations with the research team on related topics will be contacted by email to ask if they have any literature on the topic, specifying our inclusion criteria. However, in this category of publication, owing to limited resources, only publications written in French, English or Italian will be included. The choice of Italian is explained by Italy’s advances in the field of ICC [21,22] but also by the fact that one of the researchers understands this language.

#### Data management

We will export the citations identified from different electronic bibliographic databases to Zotero software. These citations will be merged and the duplicates will be identified and removed. Then, we will consider the unique citations for the selection process.

### Selection of studies

Studies will be selected independently by two members of the research team. The purpose of this parallel work is to lower:

- the risks of excluding relevant publications (studies) or including others that are not relevant;
- the risks of error of judgment and subjectivity that may result from a selection made by a single researcher.

This epistemological consideration is intended to ensure the reproducibility of the study selection process (inter and intra-researcher), traceability and transparency [23]. A selection grid based on inclusion and exclusion criteria will be designed and tested to guarantee two reviewers’ common understanding of the inclusion and exclusion criteria. The final selection grid will be documented in the appendices of the final report.

In addition, the study selection process will include three main steps and will be conducted according to the methodological standards for producing systematic reviews advocated by Cochrane [23].

#### Preliminary selection

Based on the unique citations identified, each of the two researchers will systematically apply the main inclusion criteria when reading titles, and abstracts. When multiple publications report different results on the same sample or the same results on a larger sample or results at two different points in the study, these publications will be treated as a single study but citing all the associated references [23]. Each researcher will have to document their choices in order to ensure the transparency of this operation. In addition, at the beginning of the selection, blocks of 200 articles up to 10% of unique citations identified will be discussed by the two researchers in relation to the selection criteria in order to improve selection agreement and deepen understanding of these criteria. Literature review papers (systemic or not) and conference abstracts will be excluded if they are not accompanied by papers on empirical studies about the same intervention approach.

#### Comparison of the two researchers’ results

For studies with conflicting selections, titles and abstracts will be discussed by both researchers. For results giving rise to questions, these citations will be considered for the next step of selection, that is, the complete reading of articles. In the end, a list of studies deemed relevant for full reading will be drawn up.

#### Complete reading of the selected writings and appraisal of their actual relevance

At this stage, a complete reading of the selected studies will be carried out using the selection grid (Appendix 1). Studies that do not meet the inclusion criteria will be excluded. Those that have raised questions or doubts and for which the research team and the advisory committee were unable to include or exclude will be set aside and their authors will be contacted to obtain additional information to allow a decision to be made. Studies whose authors have not responded to requests within the two-month deadline will be excluded for lack of information, despite the presumed potential for relevance. A provisional list of selected studies will be developed and will include an explanatory section on the reasons for exclusion.

Finally, a flow chart will be produced according to the PRISMA (Preferred Reporting Items for Systematic Reviews and Meta-Analyses) [23] and will be presented in the final report.

#### Judgment on the rigour of selected writings

Subsequently, the quality of the provisionally selected studies will be assessed using the Mixed-Method Appraisal Tool (MMAT), [24]. This tool is suitable for realist synthesis given its flexibility, including qualitative, quantitative and mixed-methods empirical studies.

### Data extraction

The data will be extracted from the full text of each study and any additional information provided by the authors if needed. We will develop and test a data extraction grid and its codebook describing the names, types, definitions and characteristics of selected variables. Through these elements, we will characterize the PICOSS elements as well as the CMOc elements. The grid and codebook will be developed consulting our content experts and using relevant information from the Cochrane data extraction grid [23]. Following is a preliminary list for the variables of interest:

- **Study characteristics:** paper title, study country, source (data base, snowball strategy), category of research (qualitative study, mixed study, etc.)
- **Population characteristics:** the entire community; an economically disadvantaged group (economic or material poverty), a socially vulnerable group (single, divorced, widowed, single parents with or without dependent children, etc.), a cultural or ethnic group (immigrant or Aboriginal community, etc.), families with multiple needs, etc.
- **Context characteristics:** general context of the intervention (political support, institutional framework, degree of openness and acceptance of the environment where the intervention takes place, social and cultural influences, etc.) and specific context of the intervention (knowledge of the local area and its population, interdisciplinary collaboration, cross-sectoral partnership, organization of health care and social services, coordination of practices, citizen empowerment, etc.).
- **Intervention characteristics:** intervention approach (localized community health care, localized community social care, localized and integrated community health care and social care, etc.); intervention and management strategies.
- **Outcome characteristics:** improving health and well-being of individuals and communities, health equity, social capital within the community, social networks, social cohesion, and participation in co-production; improving accessibility, availability and continuity of health care and social care; taking action on social determinants of health, etc.
- **Characteristics of different causal links describing the mechanisms studied:** logical sequencing between the element identified as a mechanism associated with the intervention strategy (actors’ reasoning and reaction to the resources of the intervention), effect trigger (generated by the mechanism) in a given context (need to be met or responded to, problem to be solved or situation to be improved).

Two researchers will independently pilot the data extraction using two studies to ensure a common understanding of the use of the extraction grid between extractors and the precision of variables of interest. The results of this piloting will be discussed with the team members and the data extraction grid will be readjusted if necessary. After conclusive piloting, the two researchers will independently extract data from the included studies. Disagreements will be discussed between the extractors and the third researcher will make the final decision when the consensus is not reached. We will contact the corresponding authors of studies via email when relevant information is missing or unclear.

Nuggets of evidence [25] will be searched for in the extracted data to illustrate the theoretical components of rough theory. These nuggets of evidence will be analyzed and compared to the rough theory in order to derive demi-regularities in the form of CMO configurations. CMOc will be discussed with the research team and the advisory committee and their relevance will be analyzed. Different CMOc will be tested for their relevance related to the research question.

## Middle-range theory development

Middle-range theories will be developed by the research team and will be improved by discussing with the advisory committee.

### Initial rough theory development

**Figure 2:**
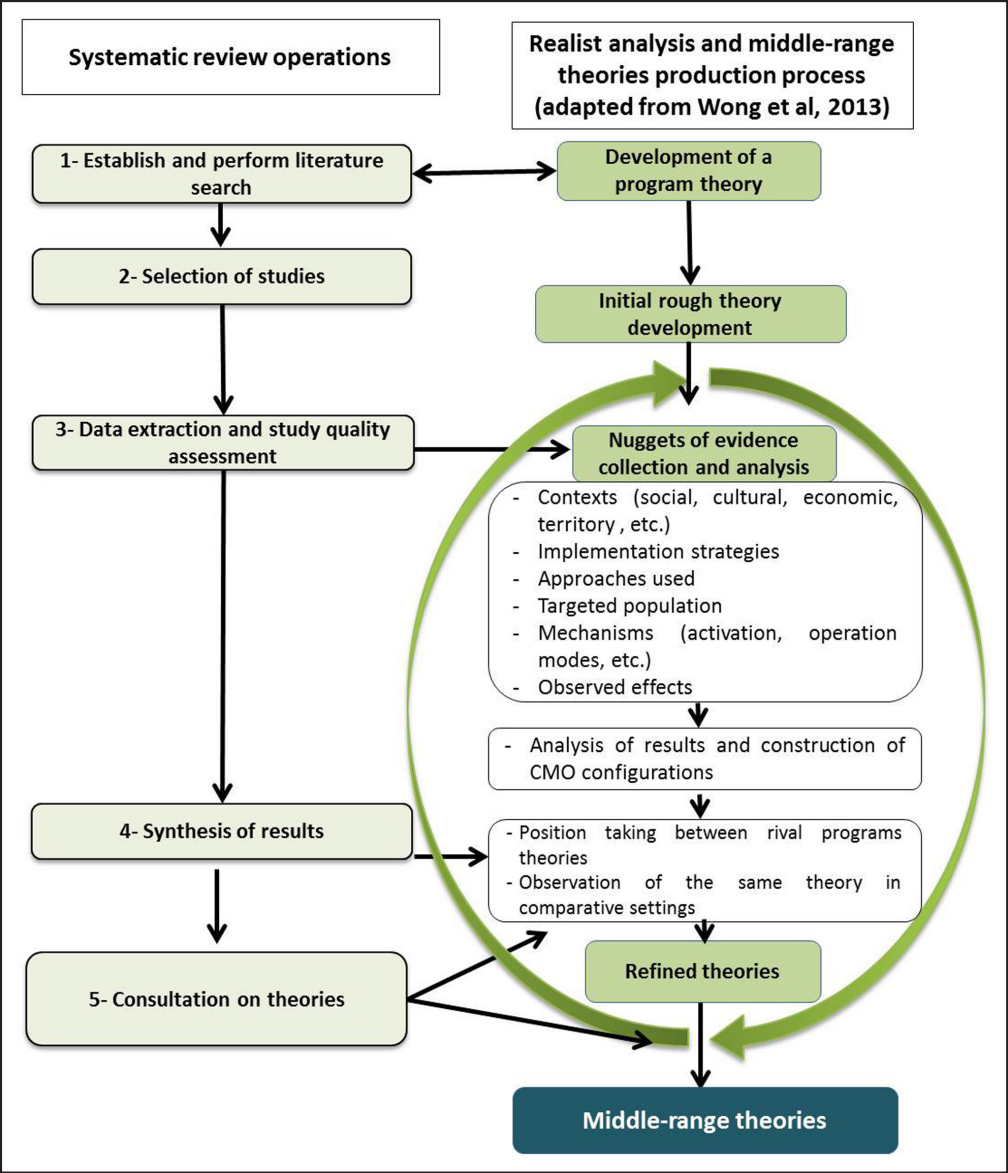
Steps in conducting the realist synthesis and process for producing the middle-range theory(ies).

Concurrently, a rough theory on the functioning of ICC will be developed in order to better understand their implementation processes, the mechanisms that can be activated by the stakeholders and the process of producing effects. This rough theory will be designed in the form of a realist program theory [26,27,28]. The theory will be developed from documents produced by our team on ICC practices and will undergo an iterative process of refinement as the research progresses (***Figure 2***). The team engaged in the process will be researchers with experience in realist evaluation and program design and evaluation. Two team members will develop the first draft and split the theory according to the two main stakeholders engaged in ICC: care workers and managers. Research team members will contribute to the discussion from their expertise point of view and finally, the advisory committee will have a meeting on rough theory to contribute to its development.

### Data synthesis

In a realist approach, the synthesis stage is an opportunity to gradually highlight the theories that support interventions [8]. Two realist synthesis strategies will be combined.

#### Synthesis for taking positions between rival theories

CMO configurations will be analyzed and compared to each other [29]. The research team and the advisory committee will take a position on the competing CMO configurations and the most appropriate one will be selected. It should be noted, however, that this first synthesis strategy does not concern the effectiveness of ICC, but only the explanation of how it works.

#### Synthesis for observing the same theory in similar contexts

This second synthesis strategy is where the effectiveness of the ICC will be analyzed [29]. The same outcomes can be measured with different tools (e.g., different Likert scales) or analysed using different formats (e.g., dichotomous, multinomial or continuous formats). Therefore, we will standardize intervention effects on outcomes of interest using the appropriate transformation methods proposed by Borenstein et al. [30] when possible. This will facilitate the possible comparisons and ICC effect pooling. Moreover, since we anticipate that most studies on ICC are qualitative studies, we plan to use a saturation approach ensuring that more frequent and precise outcomes are central to synthetized CMOc and the middle range theory.

- Analysis of how and why some ICC interventions operate in some contexts and not in others.
- Analysis of the way in which their effects derive from the contexts in which they are deployed, but above all from the mechanisms implemented by the actors.

Subsequently, mapping software will be used to design a map to visualize the causal links between the ICC interventions that are working, the approach, the environments in which they are deployed, the targeted populations and the effects produced. The analysis of this mapping will help collect the information needed to refine the rough theory (***Figure 2***). The entire research team and the advisory committee will be part of the synthesis.

### Production of one or more middle range theories

The rough theory thus refined and the CMO configurations on which it is based will be presented to localized local social care or health care teams and to users of these services with the goal of improving them in relation to actual practices. Subsequently, a middle-range theory will be produced to facilitate understanding of how and in what context the mechanisms of the stakeholders make the ICC operate and produce the anticipated outcomes.

## Dissemination of results

In the last decade, different health and social care systems have been integrated in huge public institutions and distanced from the population and the local area. Consequently, the results of the realist synthesis will help to illustrate the relevance of ICC in terms of bringing services closer to the population in a meaningful way. The findings of the present realist review will be useful for different stakeholders such as social and health system managers and policy-makers, social and healthcare professionals, citizens, and researchers. Therefore, we will use different channels and knowledge products to disseminate our findings in order to reach each category of stakeholders. Dissemination of results will be done through academic publications, presentations and a policy brief. The brief will include evidence on how ICC can be deployed by public health care and social care networks to produce the outcomes targeted by these practices, i.e., improving the health and well-being of individuals and communities, health equity, social capital in the community, social networks, social cohesion, and participation in co-production. The presentations will be adapted and accessible to different categories of stakeholders. The presentations will be held online or face-to-face, to maximize dissemination and knowledge transfer. Specifically, in the context in which the authors of the paper evolve, they are participating in the drafting and dissemination of a national public policy on the territorialisation of health and social care services. The realist synthesis will be use to illustrate how ICC operates in different context while disseminating this national strategy.

## Limits to the realist synthesis

The first limitation to the realistic synthesis the limited information available in scientific papers. Research on ICC is becoming more frequent, but relatively recent in our knowledge of the subject. The outcomes identified in the literature are mostly limited in time and clear impacts are yet to be identified, although the case of the micro-area intervention of Trieste, Italy, is instructive in this respect. Synthesizing the evidence to identify CMOc’s will require extensive deliberation among members of the research team and wider consultation to ensure that the middle range theory identified is relevant to both research and practice.
